# 591 Efficacy of a 3D Printed Microstomia Orthosis on Mouth Opening and Function, a Case Series

**DOI:** 10.1093/jbcr/irae036.225

**Published:** 2024-04-17

**Authors:** Zoë Edger-Lacoursière, valérie Calva, Genevieve Schneider, Bernadette Nedelec

**Affiliations:** Villa Medica Rehabilitation Hospital and McGill University, Montréal, QC; Villa Medica Rehabilitation Hospital, Montréal, QC; Villa Medica Rehab Center, Montréal, QC; McGill University, Montreal, QC; Villa Medica Rehabilitation Hospital and McGill University, Montréal, QC; Villa Medica Rehabilitation Hospital, Montréal, QC; Villa Medica Rehab Center, Montréal, QC; McGill University, Montreal, QC; Villa Medica Rehabilitation Hospital and McGill University, Montréal, QC; Villa Medica Rehabilitation Hospital, Montréal, QC; Villa Medica Rehab Center, Montréal, QC; McGill University, Montreal, QC; Villa Medica Rehabilitation Hospital and McGill University, Montréal, QC; Villa Medica Rehabilitation Hospital, Montréal, QC; Villa Medica Rehab Center, Montréal, QC; McGill University, Montreal, QC

## Abstract

**Introduction:**

Microstomia, often observed following facial burn injuries, can hinder functional activities such as eating, speaking, dental care, and medical procedures such as intubation. Thus, microstomia orthoses (MO) are used to increase mouth opening and function. Various designs of custom fabricated MOs have been published, however, to the best of our knowledge, the first report of a 3D MO for mouth burns was presented at the 2023 ABA conference. This 3D MO was modified to generate additional sizes and address issues of comfort and material rigidity. The modified 3D MO (available on Thingverse) was then trialed with 3 patients at different stages of their rehabilitation and their outcomes documented.

**Methods:**

The purpose of this case series was to evaluate the efficacy of a 3D MO on mouth opening and function when used to treat 3 adult burn survivors. The mouth impairment and disability assessment (MIDA), including measures of horizontal and vertical mouth opening, was completed before the use of the 3D MO and after 4 months of wear. All participants had previously reached a plateau on the MIDA and opening measures after the use of various commercially available MOs before starting the 3D MO. They were also asked to evaluate the comfort and whether they would modify any aspect of the MO.

**Results:**

The total material cost of this MO ranged from 2-3 $CAD. The print time ranged between 5.5-8 hrs (depending on mouthpiece size) using a 3D printer valued at ~250$CAD. The 3 participants were male with a mean age of 40 years, mean TBSA of 9.5% and 2/3 participants received face grafts. Patients started using the 3D MO at different timepoints post-burn: 4m, 9m and 15m. The 4-month post 3D MO wear MIDAs are currently being completed. However, one patient completed the questionnaire, which improved by15 points. Another patient was able to avoid having reconstructive surgery. To date, all patients improved both their horizontal and vertical mouth opening measures (mean improvement - horizontal: 0.72 (0.52) cm range [0.16-1.2]; vertical: 0.92 (0.41) cm range [0.67-1.4]). The MOs were worn between 2 weeks-4 months for a minimum of 30 min/day to achieve these results, but data collection is ongoing. All patients reported that the MO was comfortable, and they prefer it to all the MOs they had worn previously.

**Conclusions:**

Although data collection is ongoing, patients who had previously plateaued have improved function and oral opening after the application of this modified 3D MO.

**Applicability of Research to Practice:**

This cost-effective solution increases accessibility of MOs for individuals who do not have access to commercially available MOs. The need for economically feasible alternatives is particularly important in developing countries where burn injuries are more prevalent.

